# A Step into Medicine for New Silver(I) Complexes—From Antimicrobial Properties to Regenerative Potential

**DOI:** 10.3390/ijms27167167

**Published:** 2026-08-11

**Authors:** Kseniya A. Koshenskova, Katia Barbaro, Inna V. Fadeeva, Fedor M. Dolgushin, Lada S. Razvorotneva, Ekaterina A. Samoilenko, Maria A. Teplonogova, Irina L. Udyanskaya, Veronica Manescu (Paltanea), Iulian Vasile Antoniac, Igor L. Eremenko, Julietta V. Rau, Adam Razvan, Irina A. Lutsenko

**Affiliations:** 1 N. S. Kurnakov Institute of General and Inorganic Chemistry of the Russian Academy of Sciences, Leninsky Prosp. 31, 119071 GSP-1 Moscow, Russia; ksenia-18.11.99@mail.ru (K.A.K.); fmdolgushin@gmail.com (F.M.D.); ladanichegoneznau@mail.ru (L.S.R.); samoilenko2113@gmail.com (E.A.S.); m.teplonogova@gmail.ru (M.A.T.); ileremenko@yandex.ru (I.L.E.); 2 Istituto Zooprofilattico Sperimentale Lazio e Toscana “M. Aleandri”, Via Appia Nuova 1411, 00178 Rome, Italy; katia.barbaro@izslt.it; 3 A.A. Baikov Institute of Metallurgy and Material Science, Russian Academy of Sciences, Leninsky 49, 119334 Moscow, Russia; fadeeva_inna@mail.ru; 4 Institute of Pharmacy, Department of Analytical, Physical and Colloid Chemistry, Sechenov First Moscow State Medical University, Trubetskaya St. 8, Build. 2, 119048 Moscow, Russia; udyaskaya_i_l@staff.sechenov.ru (I.L.U.); giulietta.rau@ism.cnr.it (J.V.R.); 5 Faculty of Material Science and Engineering, National University of Science and Technology Politehnica Bucharest, 313 Splaiul Independentei, District 6, RO-060042 Bucharest, Romania; veronica.paltanea@upb.ro (V.M.); antoniac.iulian@gmail.com (I.V.A.); 6 Faculty of Electrical Engineering, National University of Science and Technology Politehnica Bucharest, 313 Splaiul Independentei, District 6, RO-060042 Bucharest, Romania; 7 Academy of Romanian Scientists, 54 Splaiul Independentei, RO-050094 Bucharest, Romania; 8 Istituto di Struttura della Materia, Consiglio Nazionale delle Ricerche (ISM-CNR), Via del Fosso del Cavaliere 100, 00133 Rome, Italy; 9 Orthopedic Department, Clinical University Emergency Hospital Elias, 17 Maraști Blvd., 011461 Bucharest, Romania; 10 Faculty of General Medicine, George Emil Palade University of Medicine, Pharmacy, Science and Technology of Targu Mures, 38 Gh. Marinescu Street, 540142 Targu Mures, Romania; 11 Research Institute of Molecular and Cellular Medicine, Peoples’ Friendship University of Russia International denomination (RUDN), 6 Miklukho-Maklaya St., 117198 Moscow, Russia

**Keywords:** silver(I) compounds, crystal structure, antimicrobial properties, cell proliferation, biocompatibility

## Abstract

Silver complexes are potential antimicrobial agents that trigger destructive processes in bacterial cells, leading to their death. Their ability to simultaneously stimulate tissue regenerative properties also makes them promising components in the development of medical materials. A common complication arising from the treatment of bone tissue damage is hospital-acquired infection, requiring repeated revision surgeries. The use of bone grafts with antibacterial properties can help solve this problem. New pyridine complexes of silver(I) with thiophenecarboxylic (Htph) and nitric acid anions have been synthesized: [Ag(tph)(py)_2_]_n_·nHtph (**1**, py—pyridine) and [Ag(bpy)]_n_·nNO_3_ (**2**, bpy—4,4′-bipyridine), the structures of which have been determined using X-ray analysis. Despite the fact that **2** is a known structure, we were able to obtain new cell parameters by conducting an X-ray diffraction analysis at a lower temperature of 100 K. According to X-ray diffraction data, both compounds are polymers in which the cationic part is formed by linear fragments (CN_Ag_ = 2) [Ag(py)_2_]^+^ (**1**)/[Ag(bpy)_2_]^+^ (**2**), and acid anions act as counterions. The presence of intermolecular and non-valent π-π interactions provides additional stabilization of supramolecular levels. Aqueous solutions **1** and **2** are stable for two weeks, according to UV-Vis data. Incubation of crosslinked resorbable polymer matrices functionalized separately with complex **1** or **2** in saline solution revealed a sustained, prolonged release of silver ions over 14 days. Evaluation of the antimicrobial potential of **1** and **2** against Gram-positive/Gram-negative species and fungi demonstrated moderate, controlled bacteriostatic growth inhibition broadly across tested strains, confirming the bifunctionality of the crosslinked hybrid matrix systems—moderate suppression of bacterial growth while simultaneously stimulating tissue regeneration. An increase in cell viability, expressed in their active proliferation under the influence of complexes **1** and **2** up to 156%, as well as calcium deposition, indicates the high cytocompatibility and pro-mineralization capacity of the new compounds.

## 1. Introduction

The development of functional biomaterials with preventative antimicrobial activity is a socially significant task. In many areas of surgery and implantology, the use of topical antibiotics (e.g., in implants and wound dressing) is impossible, and achieving optimal concentrations through systemic therapy leads to the development of toxic effects. Furthermore, systemic antibiotic administration often fails to prevent localized bacterial colonization on implant surfaces and biomaterial interfaces, where early biofilm formation can lead to persistent hospital-acquired infections. The use of specific antibacterial agents is particularly limited due to the risk of cumulative cellular and organ toxicity [1]. An alternative to antibiotics is the use of agents with antimicrobial activity, such as coordination compounds containing bactericidal ion components, particularly Ag^+^. Silver complexes, once inside a bacterial cell, trigger a cascade of destructive processes: they disrupt the membrane structure and permeability, bind to DNA, block the function of vital enzymes (glutathione reductase, thioredoxin reductase, and dehydrogenase), and cause oxidative stress. This generally reflects a multimodal approach and opens up broader possibilities for a combination therapy strategy than antibiotics (including the difficulty of bacterial cell resistance) [2,3,4]. This activity is largely determined by the availability of silver in the cell’s biological environment. For a long time, the poor solubility of Ag-based antibacterial biomaterials was an obstacle to the creation of silver-based agents. Therefore, the main developments were conducted (and continue to be conducted) on more accessible forms for research based on colloidal silver, silver oxide, submicro or nanoparticles [5,6,7,8]. However, such components have a number of drawbacks, primarily associated with silver oxidation, which leads to a deterioration in pharmacological characteristics. In this regard, silver complexes are more promising agents than silver oxide or submicro or nanoparticles [9,10,11,12].

Modern research is aimed at creating not only effective molecules, but also stable and bioavailable forms of silver compounds. Among the well-known silver preparations, silver sulfadiazine, silver nitrate, and silver thiosulfate can be distinguished [13,14]. Previously [15,16,17,18,19], we synthesized silver(I) complexes and studied their antimycobacterial properties against *Mycolicibacterium smegmatis* (a model non-pathogenic strain used for primary anti-tuberculosis evaluation). In all studied complexes, their activity was comparable to or higher than the reference drug *Rifampicin*.

Regarding orthopedics and active wound management, incorporating biologically active coordination complexes into functional biomaterials has become essential for successful bone tissue engineering and protective surface coatings. Modern regenerative medicine has recognized that merely providing structural support is not enough to repair complex bone defects; effective implants must interact favorably with the surrounding cellular microenvironment. This requires a careful balance between osseoconductivity—often provided by biocompatible polymer matrices such as polyvinylpyrrolidone and sodium alginate (PVP/ALG)—and the targeted delivery of therapeutic agents to support cellular growth and extracellular matrix deposition while conferring localized surface protection against bacterial adherence.

However, using silver-based agents in orthopedic and tissue engineering biomaterials poses significant challenges. The main difficulty is the narrow therapeutic window of silver: while high concentrations are necessary to achieve rapid bactericidal clearance, they can also lead to serious cytotoxicity. This cytotoxicity can inhibit the proliferation of mesenchymal stem cells and impair subsequent tissue regeneration. To overcome this challenge, it is crucial to precisely control the release kinetics of active silver species. In the context of localized tissue scaffolds, providing controlled, bacteriostatic surface protection that inhibits initial bacterial colonization and biofilm formation—rather than high-dose systemic bactericidal clearance—is critical to preserving host cell viability. While low-dose exposure to controlled silver species has been hypothesized in the literature to stimulate mild cellular responses (often discussed in the context of hypothetical hormetic pathways), maintaining localized, non-cytotoxic concentrations is essential to preserve cell viability while supporting cell proliferation and matrix mineralization.

In the present work, we continued to study the prospects of silver(I) complexes with antimicrobial activity. The coordination compounds [Ag(tph)(py)_2_]_n_∙nHtph (**1**) and [Ag(bpy)]_n_∙nNO_3_ (**2**) were obtained, and their structure was established directly by X-ray analysis. The stability of the obtained complexes **1** and **2** in water was studied using UV-Vis spectroscopy to confirm structural integrity during solution-state processing and scaffold manufacturing. The moderate bacteriostatic properties of crosslinked PVP/ALG matrices functionalized separately with complex **1** or complex **2** against Gram-positive and Gram-negative species as well as fungi, alongside their cytocompatibility and pro-mineralization potential, were assessed.

## 2. Results and Discussion

### 2.1. Synthesis and Single Crystal X-Ray of ***1***, ***2***

**Figure 1:**
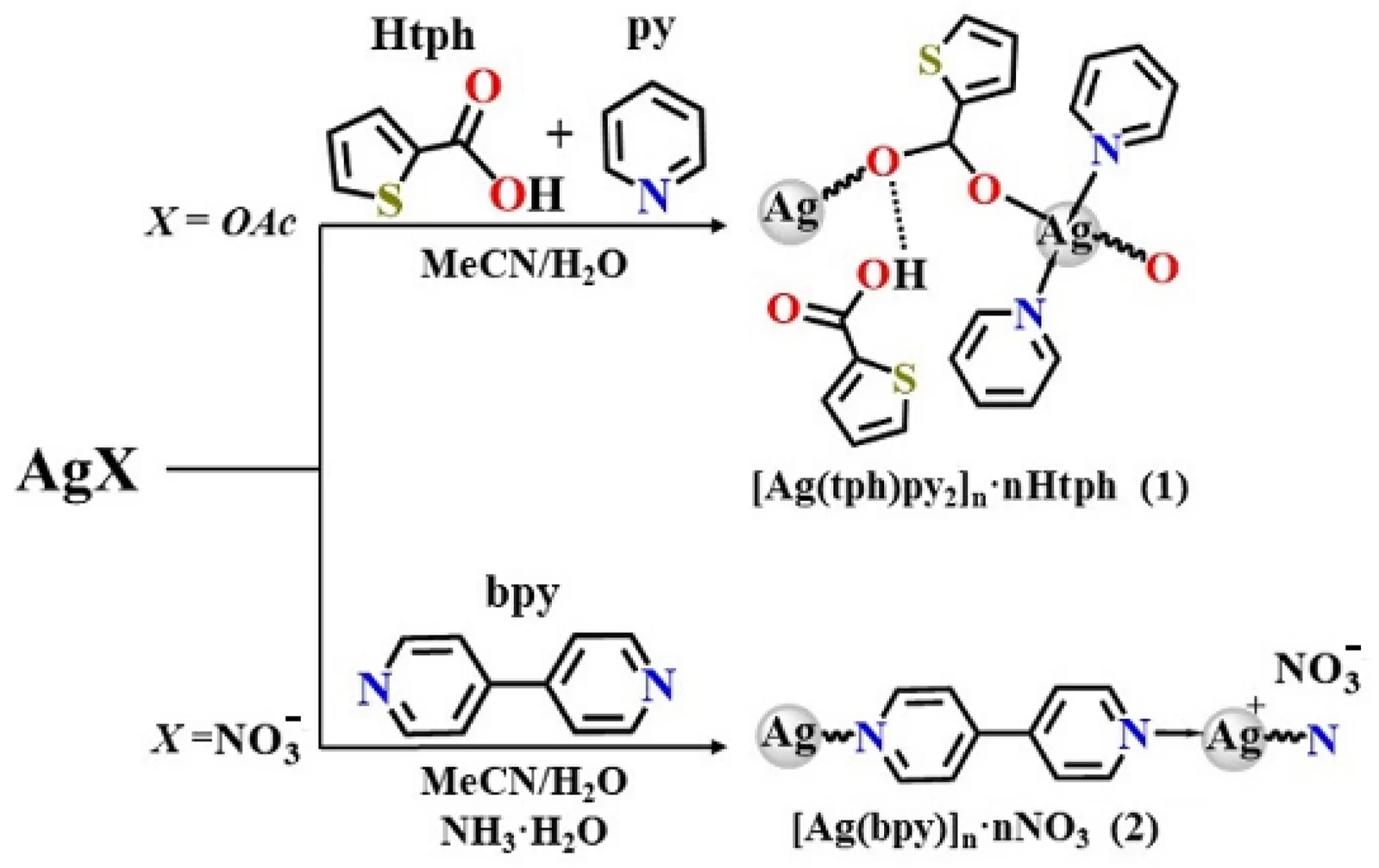
Synthetic ways for obtaining complexes **1** and **2**.

Coordination compounds **1** and **2** were obtained from silver(I) salts under mild conditions. In the case of complex **1**, silver(I) acetate was used to carry out an exchange reaction with the 2-thiophenecarboxylic acid anion, followed by the addition of pyridine as an auxiliary N-donor ligand. The reaction was carried out in a solvent system of acetonitrile—water (1:1), and crystals suitable for X-ray crystallography were obtained by slow evaporation of the solvent. Importantly, the choice of the acetate starting material facilitates the in situ deprotonation of a portion of the 2-thiophenecarboxylic acid, leading to a co-crystallized system containing both the deprotonated carboxylate anion (tph^−^) and the neutral acid Htph. Complex **2** was obtained via “one pot” reaction from silver(I) nitrate and bpy also in the acetonitrile-water solvent system. Crystals suitable for X-ray crystallography were obtained after recrystallization from an aqueous solution of ammonia (Figure 1).

**Figure 2:**
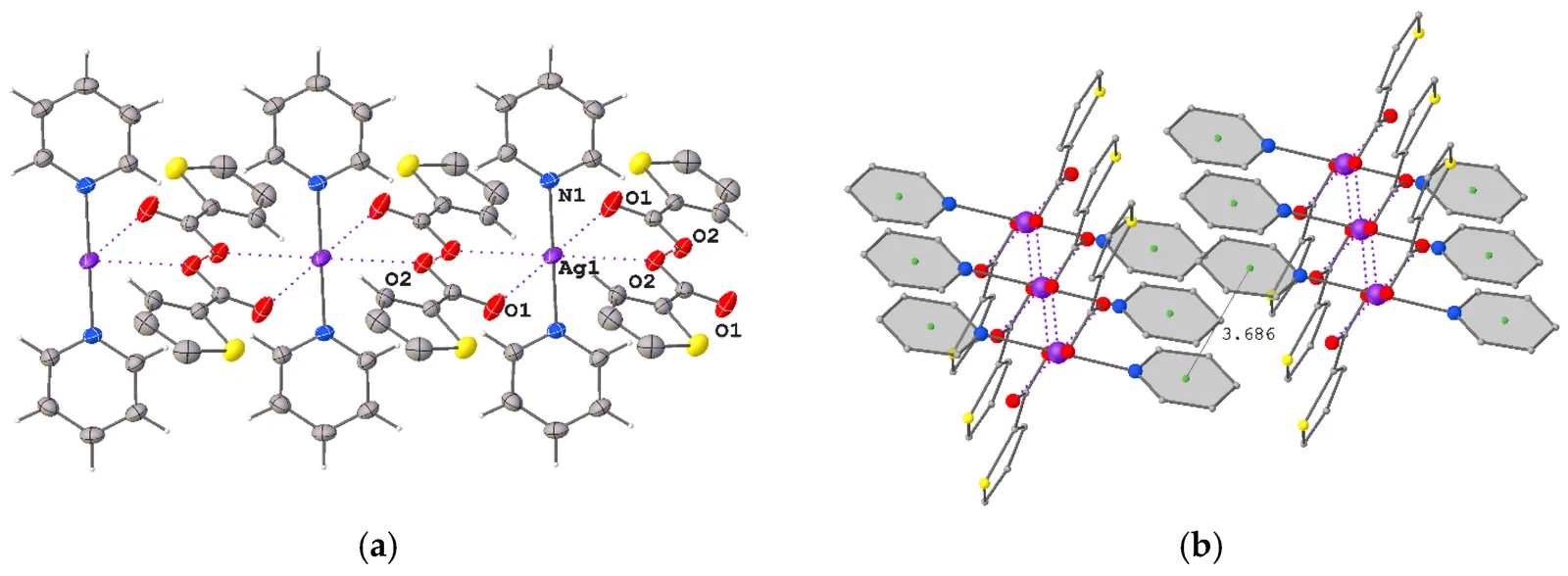
Crystal packaging of **1** (**a**); intermolecular stacking interactions in **1** (**b**) (hydrogen atoms have been omitted for clarity).

According to the single-crystal X-ray data, compound **1** crystallizes in the monoclinic space group *P*2_1_/*c* and is a one-dimensional (1D) coordination polymer. In this structure, coordination-active, positively charged fragments [Ag(py)_2_]^+^ are combined into polymeric chains due to secondary coordination interactions of the silver ions with the oxygen atoms of both the neutral Htph molecules and tph^−^ anions (Figure 2a).

Pyridine molecules are linearly coordinated to Ag^+^ (Ag(1)–N(1) = 2.203(2) Å; ∠N(1)-Ag(1)-N(1)_-x,-y,1-z_ = 180°). The complexing agent additionally participates in weak secondary interactions with the oxygen atoms of the tph^−^ and Htph anions: the Ag(1)…O(1) and Ag(1)…O(2) distances are 2.870(2) and 2.790(1) Å, respectively, which is less than the sum of the van der Waals radii of the silver and oxygen atoms (3.24 Å [20]), but exceeds the typical bond length in silver carboxylate complexes (2.3–2.5 Å; Table 1) [21,22,23]. This indicates that the primary coordination sphere of Ag(I) is dominated by the nitrogen-donating pyridine ligands, while the oxygen-containing anions support the supramolecular chain propagation via weaker, electrostatic forces. However, such weak secondary contacts with four oxygen atoms lead to a slight elongation of the Ag–N bonds in the [Ag(py)_2_]^+^ cation, compared to the same bonds 2.12–2.18 Å in the previously studied salts [Ag(py)_2_][NO_3_] ·(H_2_O) [24,25], [Ag(py)_2_][ClO_4_], [Ag(py)_2_][BF_4_], [Ag(py)_2_][PF_6_] [26].

A key supramolecular feature of **1** is the assembly of the anionic and neutral ligand components. The neutral molecule Htph and the tph^−^ anion, bound to two adjacent silver ions in the polymer chain, form a highly stable hydrogen-bonded pair due to the strong O(2)-H(2a)…O(2)_1-x,-y,1-z_ interaction. This interaction features a short O…O distance of 2.460(2) Å and an O-H…O angle of 175(5)°. Such a short donor–acceptor distance is characteristic of a strong, low-barrier hydrogen bond, which effectively stabilizes the charge-balance synthon between the neutral acid and its conjugate base within the crystal lattice. The crystal packing of 1 also contains non-valent π-π interactions between parallel aromatic rings of neighboring pyridines with a centroid-centroid distance of 3.686 Å. These stacking interactions successfully link the 1D polymer chains into supramolecular ladder layers that extend parallel to the ab plane of the crystal (Figure 2b).

**Figure 3:**
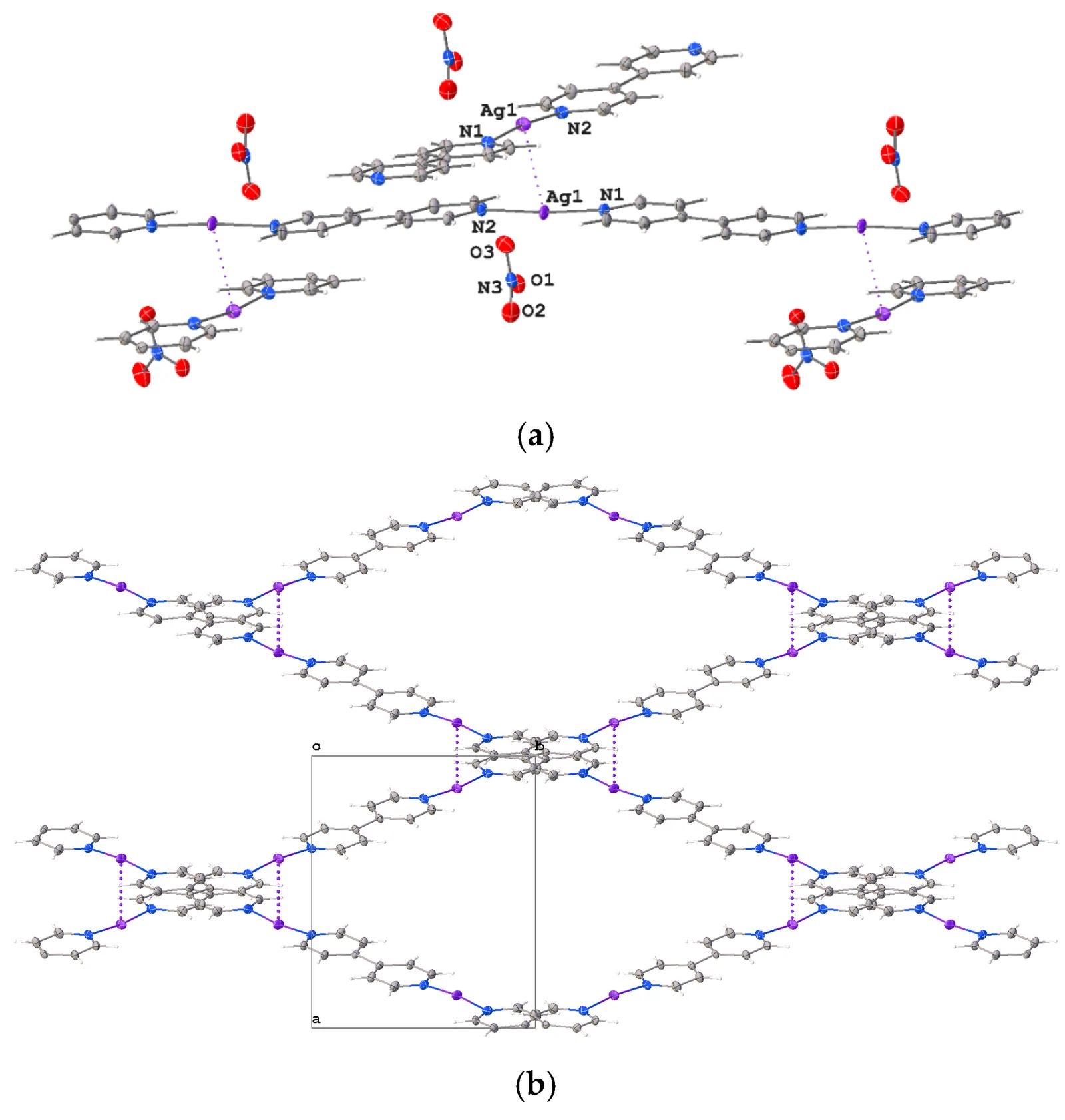
Crystal packaging of **2** (**a**,**b**) (argentophilic interactions are shown as purple dashed lines).

Complex **2** in the crystal lattice is built from cationic chains in which Ag^+^ cations linearly coordinate bpy molecules, forming an inner coordination sphere (coordination number CN_Ag_ = 2; Ag–N bond lengths of 2.140(2) and 2.144(2) Å, and a ∠N(1)-Ag(1)-N(2) angle of 173.44(9)°). The mutually perpendicular cationic chains are interlinked into a 3-D framework structure (Figure 3) primarily driven by pronounced argentophilic interactions. The Ag…Ag_-x,+y,1/2-z_ distance of 2.9539(5) Å, which is noticeably shorter than the sum of the van der Waals radii of two silver atoms (3.44 Å) [27] and only slightly longer than the interatomic bond length in metallic silver (2.89 Å). This significant metallophilic attraction plays a primary thermodynamic role in directing the perpendicular alignment of the cationic chains. In addition, the crystal structure is stabilized by non-covalent π-π interactions between the pyridine rings of bpy ligands from adjacent chains (centroid-centroid distances of 3.523 Å), as well as some weak secondary interactions between the silver ions and two of the three oxygen atoms of the nitrate anions (Table 1).

It should be noted that the unusual structure of this framework compound has been described in detail previously [28,29,30,31]. In those studies, the structure was solved in the orthorhombic space group *Fddd* (using X-ray diffraction data collected at room temperature), which exhibited disordered positions for the oxygen atoms of the nitrate anion. In contrast, the single-crystal X-ray data for complex **2** in our study were collected at 100 K. This low-temperature measurement leads to a structural solution in the monoclinic space group *C*2/*c* with a completely ordered structure. This outcome strongly points to a temperature-dependent, order–disorder structural phase transition. Upon cooling to 100 K, the thermal motion and rotational freedom of the nitrate counter-anions are restricted, locking them into a single, ordered conformation. This localized ordering induces a subtle collective shift in the framework, resulting in a symmetry reduction from the high-temperature orthorhombic phase to the more ordered low-temperature monoclinic (*C*2/*c*) phase.

### 2.2. UV-Vis Spectroscopy Studies ***1*** and ***2***

**Figure 4:**
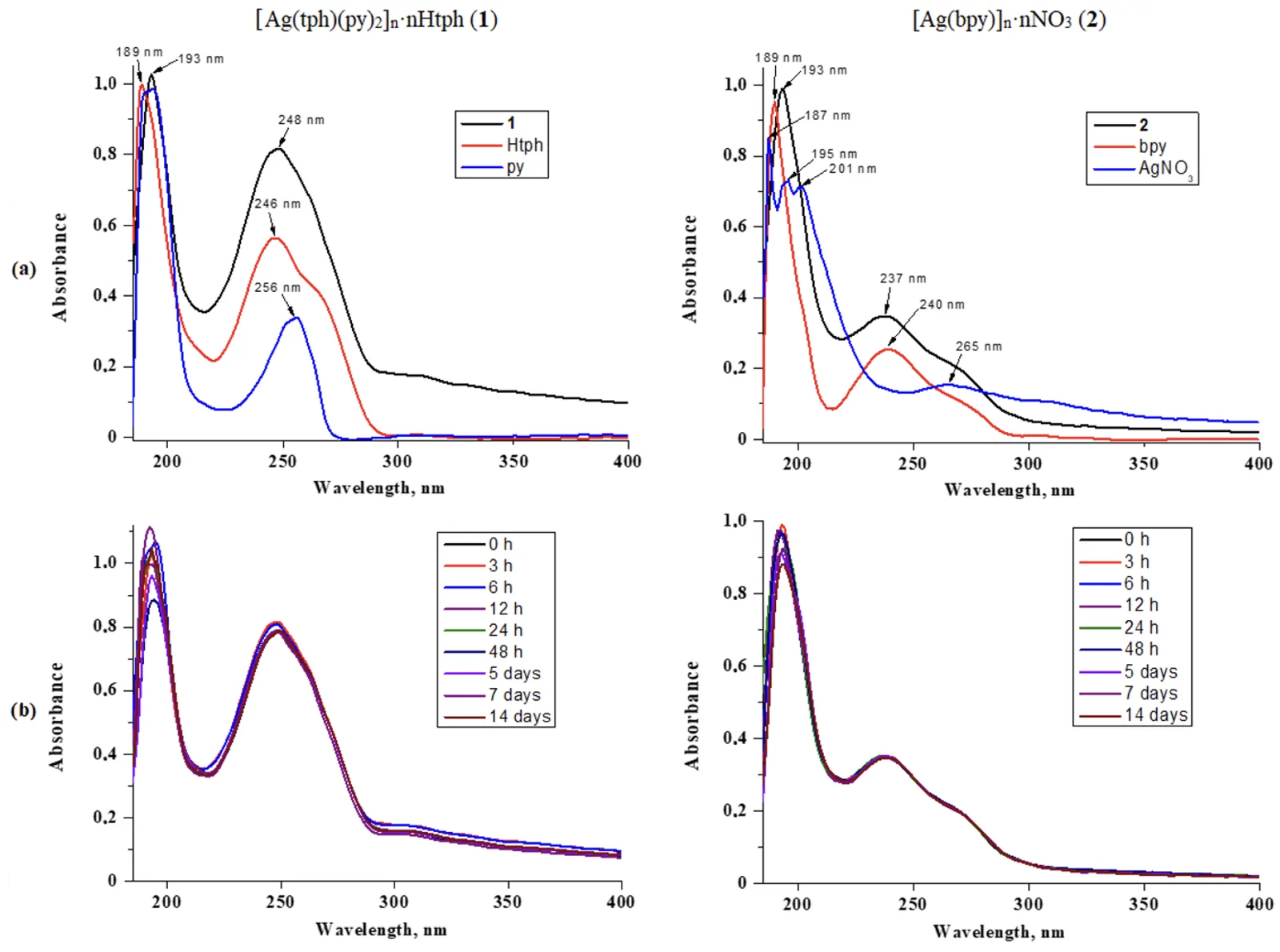
UV-Vis absorption spectra (185–400 nm) of complexes **1** and **2** in comparison with the free ligands (**a**) and over time (14 days) (**b**), recorded at room temperature in aqueous solutions at a concentration of 10^−5^ M.

The electronic absorption spectra of complexes **1** and **2** were recorded using an SF-2000 OKB spectrophotometer in aqueous solutions at a concentration of 10^−5^ M in the range of 185–400 nm. The spectra were measured at room temperature immediately after dissolution. To evaluate the electronic changes induced by coordination, a comparative analysis of the spectra of the complexes, their corresponding free ligands, and AgNO_3_ was performed. Furthermore, the stability of the compounds in solution over a period of 14 days was investigated by recording their spectra under identical conditions (Figure 4).

The spectrum of complex **1** (Figure 4a, left) exhibits an intense band at 193 nm, attributed to π → π* transitions of the aromatic systems of the thiophene and pyridine rings. A distinct broad peak is observed at 248 nm, which corresponds to the *n* → π* transitions of the ligand heteroatoms [32]. In the long-wavelength region (260–350 nm), a broad, low-intensity shoulder is observed, which can be assigned to ligand-to-metal charge transfer (LMCT) transitions Ag ← O/N [33]. Compared to the spectra of the free ligands (Htph and py), a pronounced bathochromic shift in the absorption maxima accompanied by a notable hyperchromic effect (intensity increase) is observed. These combined spectral modifications unambiguously confirm the coordination of the ligands to the Ag(I) ion [33,34].

The spectrum of complex **2** (Figure 4a, right) shows an intense band at 193 nm, assigned to the π → π* transition within the aromatic system of 4,4′-bpy. In the spectrum of the free ligand, the maximum of this band is observed at 189 nm, thus indicating a bathochromic shift upon complexation, which confirms the coordination of the ligand to the metal center. An important structural diagnostic feature is the absence of the characteristic conjugation band (B-band) at 260–280 nm, which is typically associated with the coplanar, inter-ring conjugation between the two pyridine rings in free 4,4′-bpy. Its complete disappearance in the spectrum of **2** strongly indicates a loss of this coplanarity (a rotational twisting of the rings about the central C–C bond) upon coordination to the silver(I) nodes, which is in perfect agreement with our X-ray crystallographic findings [35,36].

For both complexes, the bathochromic shift in the π → π* bands indicates an enhanced in the delocalization of the π-electrons resulting from the formation of Ag–N/O coordinate bonds [33,34]. Consequently, the presence of *n* → π* (for **1**, at 248 nm) and LMCT transitions, as well as the absence of any B-band conjugation feature for complex **2,** serve as reliable spectroscopic fingerprints of coordination, consistent with the literature data for similar silver(I) coordination networks [32,35,36,37].

The stability study (Figure 4b) demonstrates that aqueous solutions of complexes **1** and **2** are stable for at least 14 days. The overall spectral profiles, positions of the absorption maxima, and the nature of the electronic transitions remain remarkably constant over time, indicating the preservation of the metal–ligand coordination bonds in the dissolved state. The slight changes observed in the intensity of the main characteristic bands are fully attributed to minor solvent loss via evaporation, which slightly concentrates the solution over the course of the 14-day trial. These stability profiles provide crucial, independent verification of the robust solution-state behavior of complexes **1** and **2**, establishing a direct link between their solid-state crystallographic architectures and their chemical durability in aqueous media. The systematic conservation of their distinct spectroscopic fingerprints—such as the low-energy LMCT transitions for **1** and the twisted, non-coplanar conformation of the 4,4′-bpy ligands for **2**—authentically demonstrates that these coordination networks do not undergo rapid dissociation, degradation, or ligand exchange upon dissolution. Instead, they successfully retain their robust metallo-supramolecular behavior and strong non-covalent pathways under aqueous conditions. This high solution-state stability is a vital prerequisite for their potential practical applications, ranging from solution-processed material fabrication to biological and antimicrobial evaluations, where structural integrity in aqueous environments is of utmost importance. However, it should be noted that while UV-Vis spectroscopy confirms chemical stability in pure aqueous media during material synthesis and processing, biological media contain high concentrations of physiological electrolytes (such as Cl^−^ and phosphate ions) as well as thiol-rich serum proteins. Under actual physiological conditions, these components can induce competitive ligand exchange or local precipitation (e.g., as insoluble silver salt AgCl), which modulates the effective concentration of free Ag^+^ ions. Future translational studies will further evaluate the long-term chemical coordination and equilibrium of these complexes under complex simulated body fluids (SBF) and protein-rich cell culture media.

### 2.3. Preparation and Characterization of the Silver-Loaded Polymer Matrix

#### 2.3.1. Fabrication and Microstructural Architecture of the Matrix

**Figure 5:**
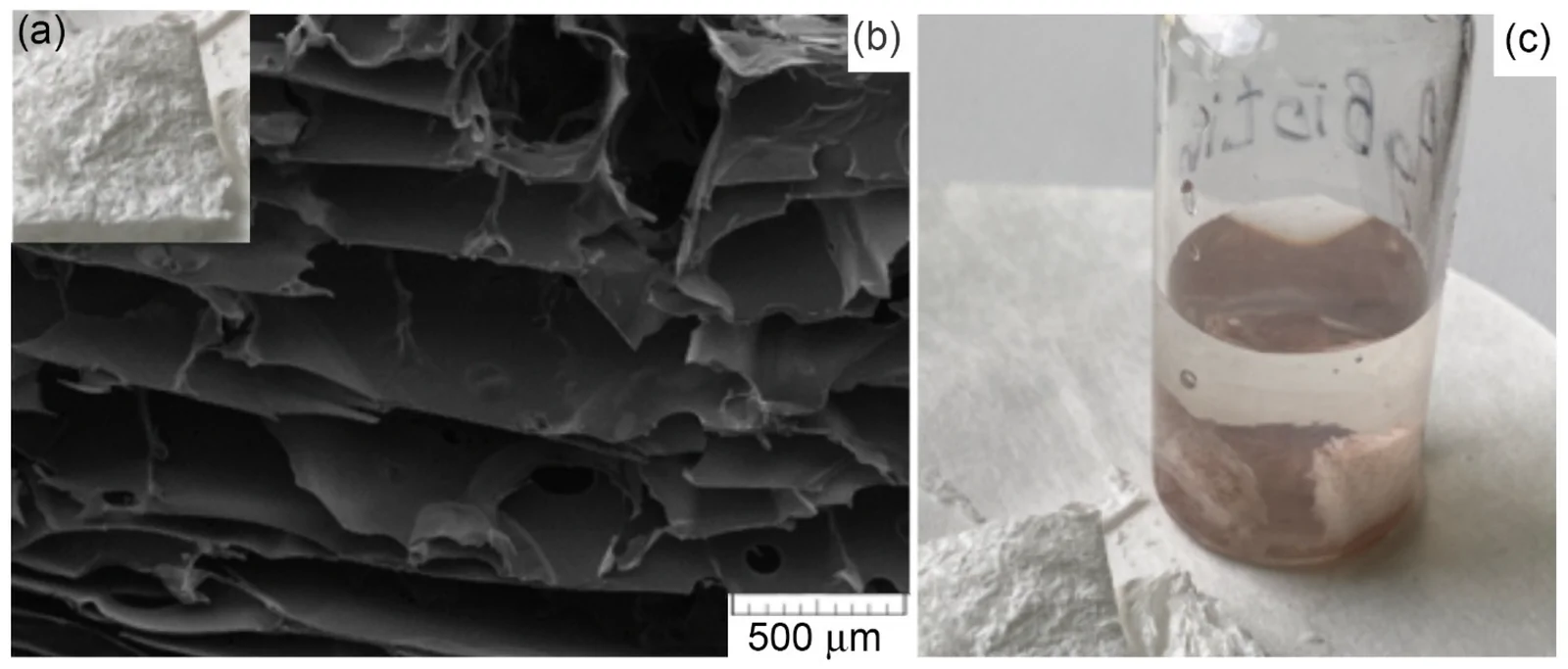
Micrographs of the matrix: (**a**,**b**)—after crosslinking with barium chloride; (**c**) matrix in solution of complex **1**.

A porous polymer matrix composed of a biocompatible blend of polyvinylpyrrolidone (PVP) and sodium alginate (ALG), blended in a mass ratio of 1:1, was used as a bioresorbable matrix to deliver the synthesized silver(I) complex to the target site (Figure 5a,c). This specific matrix was fabricated according to a protocol described in [38].

Briefly, a mixture of PVP and ALG (1:1 mass ratio) was dissolved in 97.5 mL of distilled water yielding a homogeneous 2.5% *w*/*v* polymer solution. The solution was subsequently subjected to high-shear agitation at a stirrer speed of 1000 min^−1^ until a stable foam was formed. This gaseous-liquid dispersion was then chemically stabilized by ionic cross-linking of the alginate carboxylate groups with divalent barium ions Ba^2+^ using a barium chloride solution. Following partial cross-linking, the foam was frozen at −50 °C and subsequently dried via lyophilization (freeze-drying at −50 °C under a reduced pressure of 0.05 mbar for 24 h). The microstructure of the resulting porous matrix is shown in the SEM micrograph in Figure 5a, while its behavior during immersion in the complex solution is illustrated in Figure 5b,c. The matrix exhibits a layered, cellular structure with interlamellar distances ranging from tens of microns to 300 μm and a wall thickness of up to 10 μm. This highly porous, hierarchical network is critical, as it provides a high specific surface area that facilitates efficient solvent capillary action and subsequent ligand-metal diffusion. The dried porous matrix was immersed in a solution of the respective silver complex for 30 min to allow complete wetting, followed by drying in air at room temperature.

#### 2.3.2. In Situ Nanoparticle Formation and SEM Analysis

**Figure 6:**
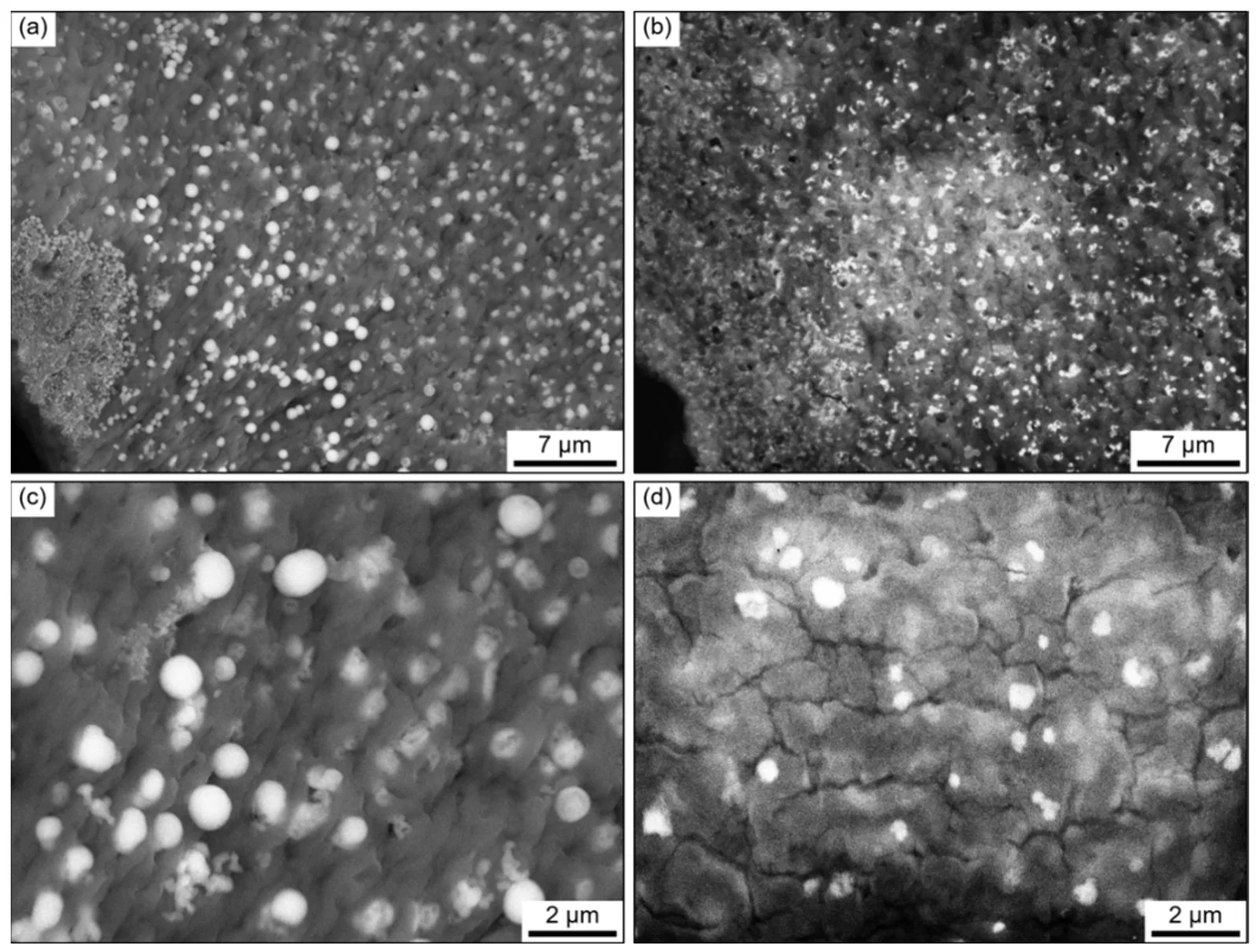
SEM images of the Ag submicroparticles obtained from complex **1** (**a**,**c**) and **2** (**b**,**d**) in the polymer matrix at different magnifications.

Scanning electron microscopy (SEM) images show the presence of fine silver-containing submicroparticles within the polymer walls of both samples prepared with complex **1** and complex **2** (Figure 6). These submicroparticles are homogeneously distributed in the polymeric matrix without significant agglomeration (Figure 6a,b). Their formation is driven by the reducing properties of the polymer matrix; the hydroxyl groups of sodium alginate and the carbonyl groups of PVP act as mild, in situ reducing agents and steric stabilizers that facilitate the nucleation and growth of silver submicroparticles directly from the coordinated silver(I) precursor complexes.

However, the morphology and size distribution of the resulting particles vary significantly depending on the nature of the precursor complex used. Quantitative particle size analysis performed using ImageJ software (version 1.54, National Institutes of Health, Bethesda, MD, USA, n ≥ 100 particles per sample) indicates that the submicroparticles obtained from complex **1** have a well-defined spherical shape with a mean diameter of 420 ± 110 nm (ranging predominantly between 250 nm and 750 nm) (Figure 6c). The spherical morphology suggests a relatively uniform, isotropic growth rate controlled by the gradual release of Ag^+^ ions from the weaker coordination environment of 1. Regarding the particles obtained from complex **2**, they exhibit an irregular, non-uniform morphology with a significantly smaller mean diameter of 210 ± 65 nm (Figure 6d). This smaller size and irregular shape are attributed to the higher stability of the 3D-networked precursor **2**, which limits the rate of silver reduction and restricts growth, keeping the particles in a smaller, kinetically frozen state.

Thus, the obtained data demonstrates the formation of silver submicroparticles, which are capable of additionally providing an antibacterial effect.

#### 2.3.3. Swelling, Biodegradation, and Dual-Mode Silver Release Kinetics

**Figure 7:**
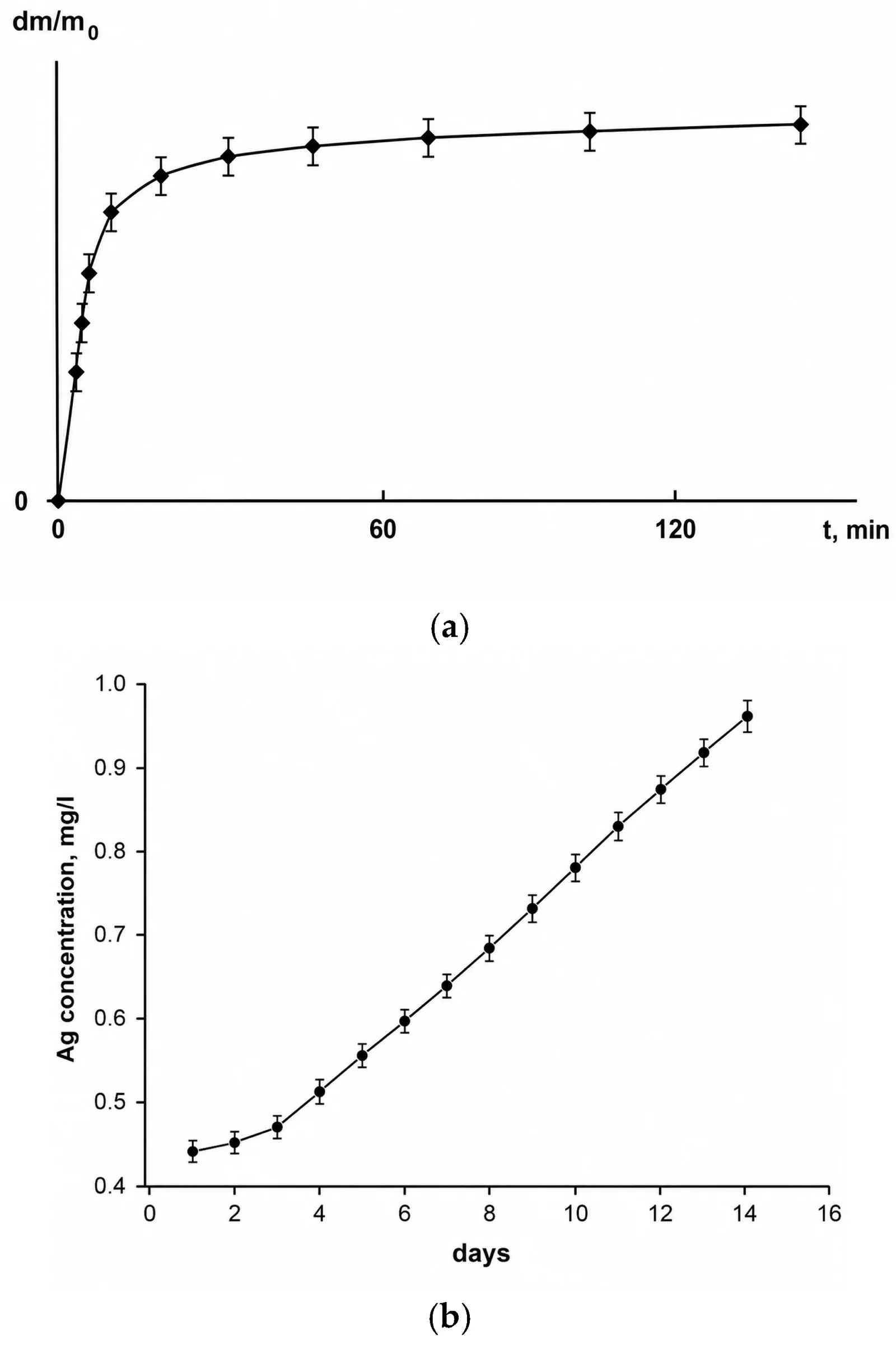
(**a**) Graph of polymer matrix swelling in saline solution during the initial 150 min (~2 h); (**b**) The concentration of Ag^+^ depending on the exposure time in saline solution. Error bars constitute 0.01 mg/L.

The swelling and erosion behavior of the carrier matrix plays a fundamental role in controlling the release kinetics of the active silver species. Upon immersion of the dry matrix in an aqueous medium, it swells rapidly during the first two hours (120–150 min), demonstrating a sharp increase in mass (Figure 7a). This initial swelling phase is driven by capillary forces and the highly hydrophilic nature of the uncross-linked PVP and ALG chains, allowing the saline solution to rapidly penetrate the interlamellar channels between the polymer layers.

**Figure 8:**
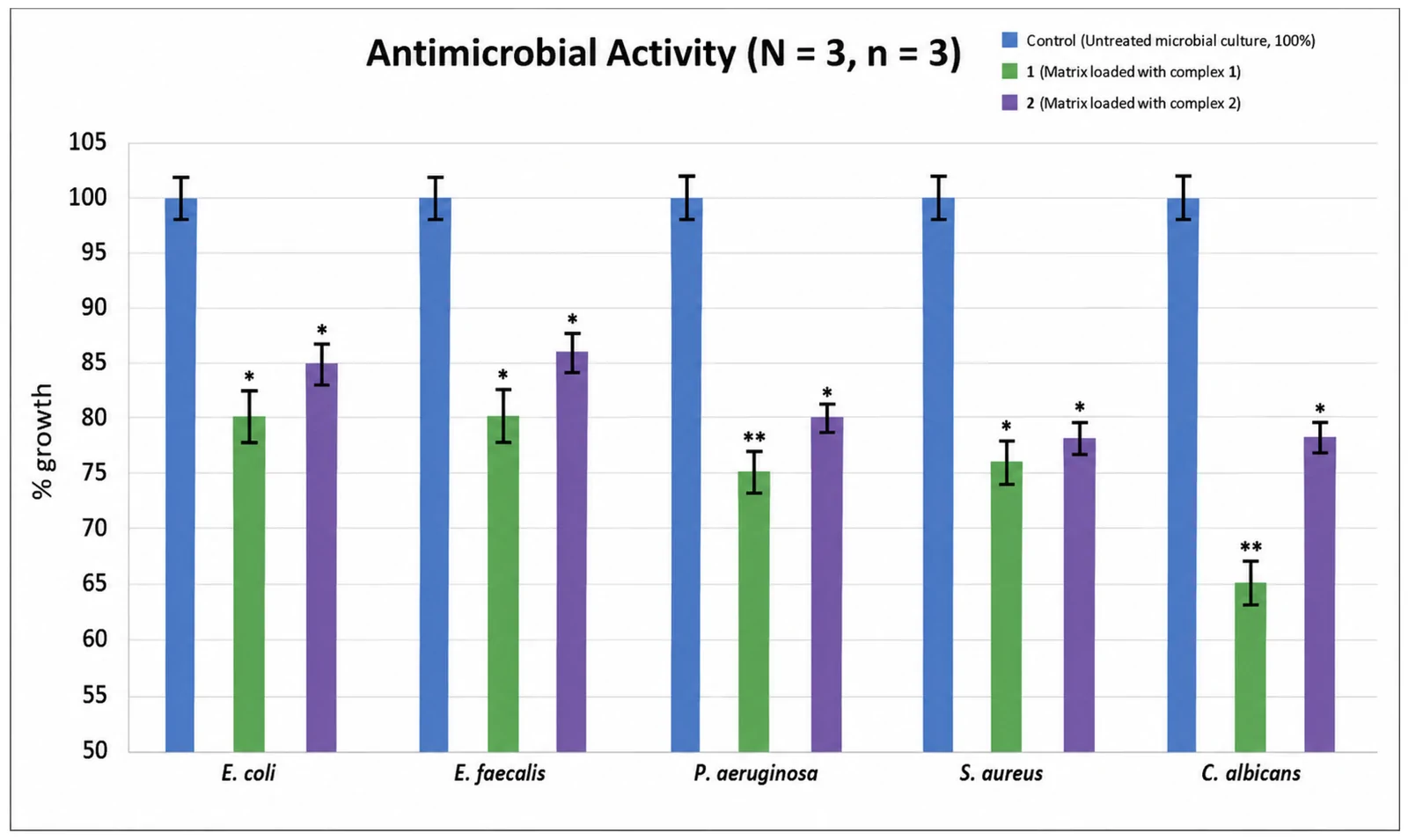
Percentage growth inhibition of microbial strains (*E. coli*, *E. faecalis*, *S. aureus*, *P. aeruginosa*, *C. albicans*) following treatment with **1** and **2** (1 mg/mL). Values are expressed as mean ± SD relative to untreated controls (100%). Statistical significance: *p* ≤ 0.05 *; *p* ≤ 0.01 **.

Following this hydration phase, when the matrix is kept in an aqueous solution for several days to several weeks, a gradual decrease in mass is observed due to the hydrolytic dissolution and macromolecular erosion of the polymer matrix [38]. Complete dissolution of the matrix occurs within 20 to 28 days, making it a highly suitable bioresorbable platform for temporary wound-dressing or tissue-regeneration applications [3,38]. To evaluate the sustained delivery profile, polymer matrices were separately loaded by immersion in solutions of complex **1** or complex **2** in DMSO for 30 min and dried in air for 24 h. The loaded matrices were then immersed in a physiological saline solution, and the cumulative concentration of silver ions released into the medium was determined after 1, 3, and 14 days (Figure 7b). The swelling, biodegradation, and cumulative ion release experiments were performed using N = 3 independent polymer matrix batches per group, with each sample measured in technical triplicate (n = 3 per batch). Data in Figure 8 represent the mean ± standard deviation (SD) derived from the N = 3 independent biological experiments. As the saline solution penetrates the hydrated polymer matrices, the loaded complexes (represented by complex **2** in Figure 7b) are steadily solvated and released into the surrounding fluid, resulting in a progressive, linear increase in the concentration of active Ag^+^ ions over 14 days [10,38]. It is worth noting that in physiological saline and biological fluids containing chloride ions, the concentration of free ionic silver is governed by a dynamic dissociation and solubility equilibrium involving localized AgCl species (K_sp_ ~ 1.8 × 10^−10^). This thermodynamic buffer helps maintain a steady, non-cytotoxic level of active silver ions in solutions.

Importantly, this delivery platform operates via a dual-mode therapeutic mechanism. The system simultaneously delivers both solid-state silver submicroparticles (embedded in the polymer walls) and dissolved silver(I) complex fragments (releasing free ionic Ag^+^). The submicroparticles provide a localized, long-term reservoir of silver, while the diffusing Ag^+^ ions offer immediate, highly bioavailable antimicrobial activity. This combination of physical and chemical silver forms in the PVP/ALG matrices establishes a highly effective synergistic antibacterial system, ensuring both rapid-onset and long-lasting protection against pathogens as the bioresorbable matrix slowly dissolves.

Consequently, the synchronized kinetics of rapid initial swelling followed by progressive matrix erosion ensure that the therapeutic silver cargo is released in a highly controlled, predictable manner, preventing toxic localized concentrations while maintaining a continuous protective barrier at the application site.

### 2.4. Antimicrobial Activity

The antimicrobial properties of **1** and **2** were evaluated against five clinically relevant microbial strains: the Gram-positive bacteria *Staphylococcus aureus* (*S. aureus*) and *Enterococcus faecalis* (*E. faecalis*), the Gram-negative bacteria *Pseudomonas aeruginosa* (*P. aeruginosa*) and *Escherichia coli* (*E. coli*), and the yeast *Candida albicans* (*C. albicans*). All antimicrobial experiments were performed using N = 3 independent biological replicates, with measurements evaluated in technical triplicate (n = 3). Data were evaluated using Dunnett’s post hoc test relative to untreated cell controls. Both silver-loaded polymer substrates demonstrated statistically significant, moderate growth inhibition across all five tested strains compared to untreated controls (Figure 8). Specifically, for *S. aureus*, substrate **1** reduced growth by 23.18% (*p* ≤ 0.05), and substrate **2** by 19.52% (*p* ≤ 0.05). For *P. aeruginosa*, substrates **1** and **2** reduced growth by 26.53% (*p* ≤ 0.01) and 19.32% (*p* ≤ 0.05), respectively. Statistically significant growth reductions were likewise confirmed for *E. coli* (19.32% for **1**, *p* ≤ 0.05; 15.75% for **2**, *p* ≤ 0.05), *E. faecalis* (19.27% for **1**, *p* ≤ 0.05; 14.57% for **2**, *p* ≤ 0.05), and the fungal strain *C. albicans* (34.70% for **1**, *p* ≤ 0.01; 22.79% for **2**, *p* ≤ 0.05). These results demonstrate a moderate yet consistent bacteriostatic capability of the hybrid polymer substrates. It should be noted that the 20–35% inhibition observed at the tested screening concentration (1 mg/mL) represents a controlled, localized bacteriostatic effect designed to mitigate early bacterial attachment and biofilm formation rather than acute bactericidal eradication. Crucially, this loading level was intentionally chosen to maintain a delicate therapeutic balance, providing localized antimicrobial surface protection without exerting cytotoxic side effects on surrounding tissue progenitor cells (as evidenced by the stem cell proliferation assays in Section 2.5).

To gain insight into the underlying biological mechanisms, it is important to analyze the observed antimicrobial activity alongside the microbial cell wall structures and the coordination chemistry of the silver complexes. For all tested strains, complex **1** consistently demonstrated a higher percentage of growth inhibition compared to complex **2**. This difference is due to their structural architectures and the respective transient release rates at which silver is liberated during initial exposure. Our release kinetics studies (Section 2.3.3) demonstrate that while long-term equilibrium ion levels in saline converge due to AgCl solubility limits (K_sp_), the short-term release behavior is governed by precursor coordination strength. The weaker secondary coordination bonds (Ag…O) in the one-dimensional chains of compound **1** are rapidly cleaved in aqueous biological media. This results in a higher initial transient burst of free Ag^+^ ions during early incubation. On the other hand, the robust three-dimensional structure of compound **2**, stabilized by strong covalent Ag–N bonds and metallophilic Ag…Ag interactions, releases Ag^+^ ions in a much slower and more sustained manner. As a result, within the timeframe of the performed microbial assays, complex **1** provides a more rapid and potent dose of active silver, leading to an enhanced bacteriostatic response.

Both complexes demonstrated broad-spectrum, relatively uniform bacteriostatic efficacy across both Gram-negative (*P. aeruginosa* and *E. coli*) and Gram-positive species (*S. aureus* and *E. faecalis*), with overall growth inhibition values remaining within a comparable range (~15–27%). This comparable level of activity indicates that the antimicrobial performance of the functionalized matrices is not strictly dependent on Gram classification. It is worth noting that both Gram-positive and Gram-negative bacterial surfaces present strongly negatively charged outer layers—governed by exposed lipopolysaccharides in Gram-negative species, and by negatively charged teichoic/lipoteichoic acids as well as carboxylate groups within the peptidoglycan matrix in Gram-positive species. Thus, rather than acting via selective surface charge attraction, the released silver species (both free ionic Ag^+^ and solvated complex fragments) operate through generalized, non-specific membrane interactions. The slight variations in sensitivity observed among individual species are likely influenced by a balance of biological factors: while the thick, highly cross-linked peptidoglycan cell wall of Gram-positive bacteria can act as a physical molecular sieve that partially slows ion diffusion toward the inner cytoplasmic membrane, Gram-negative species feature a thinner peptidoglycan layer bounded by an outer membrane where structural destabilization and periplasmic accumulation occur rapidly [39]. Overall, the dual-mode delivery of active silver species provides broad-spectrum bacteriostatic protection without relying on single-strain targeted mechanisms.

The most significant growth inhibition was observed against the eukaryotic yeast *C. albicans*, with a reduction of 34.70% for compound **1** and 22.79% for compound **2**. Fungal cell walls and membranes are primarily composed of chitin, glucans, and mannoproteins, which present a highly porous and negatively charged surface. The combination of dissolved silver complexes and embedded silver submicroparticles in the bioresorbable polymer matrices effectively interacts with these surface glycoproteins. This interaction contributes to localized membrane depolarization, increased cell envelope permeability, and impairment of fungal mitochondrial membrane potential. Additionally, compared to specific bacterial resistance mechanisms, the eukaryotic cell structure and high surface area of *C. albicans* render it particularly responsive to the synergistic dual-mode delivery of silver (both ionic Ag^+^ and silver submicroparticles) provided by these hybrid polymer materials [40].

When interpreting these antimicrobial results, several methodological aspects and media interactions must be taken into account as potential study limitations. First, the present in vitro antimicrobial screening was conducted over a standard 24 h incubation period. While this duration is ideal for evaluating early anti-adherence and initial bacteriostatic protection against primary microbial colonization, it represents an early period of the biomaterial’s lifetime performance. As shown in our extended-release kinetics (Figure 8), silver release continues to accumulate linearly over 14 days; thus, short-term 24 h assays may understate the long-term, sustained antimicrobial efficacy of the matrices. Secondly, the biological assays were carried out in BHI broth. BHI is a complex, nutrient-rich broth containing abundant peptides, proteins, and free chloride anions (Cl^−^). These components act as competitive scavengers that can rapidly bind free Ag^+^ ions (forming insoluble AgCl precipitates or organo-silver complexes), thereby lowering the effective concentration of free, bioavailable silver in the culture medium. Consequently, the observed ~20–35% growth inhibition reflects the net antimicrobial response in a highly complex biological environment and confirms that the functionalized scaffolds maintain effective bacteriostatic activity even in the presence of strong nutrient-binding competition.

In summary, these findings demonstrate that tailoring silver(I) coordination networks allows fine-tuning of their biological properties. By integrating these customizable complexes into a bioresorbable PVP/ALG polymer matrix, a functional biomaterial providing localized bacteriostatic and anti-biofilm surface protection was successfully realized. Also, long-term broth release and multi-day microbial evaluation will be set as key directions for future translational research studies.

### 2.5. In Vitro Cell Viability and Proliferation Assay (MTT)

To evaluate the biocompatibility and cytocompatibility of the silver-functionalized polymeric matrices, an MTT assay was performed using adipose-derived mesenchymal stem cells (aMSCs). This colorimetric assay directly measures mitochondrial dehydrogenase activity, serving as a reliable indicator of cell viability, metabolic state, and proliferation. All MTT assays were performed using N = 3 independent biological experiments (using distinct cell passages), with each condition evaluated in technical triplicate (n = 3). Data were statistically analyzed using one-way ANOVA followed by Dunnett’s post hoc test relative to untreated control cells.

**Figure 9:**
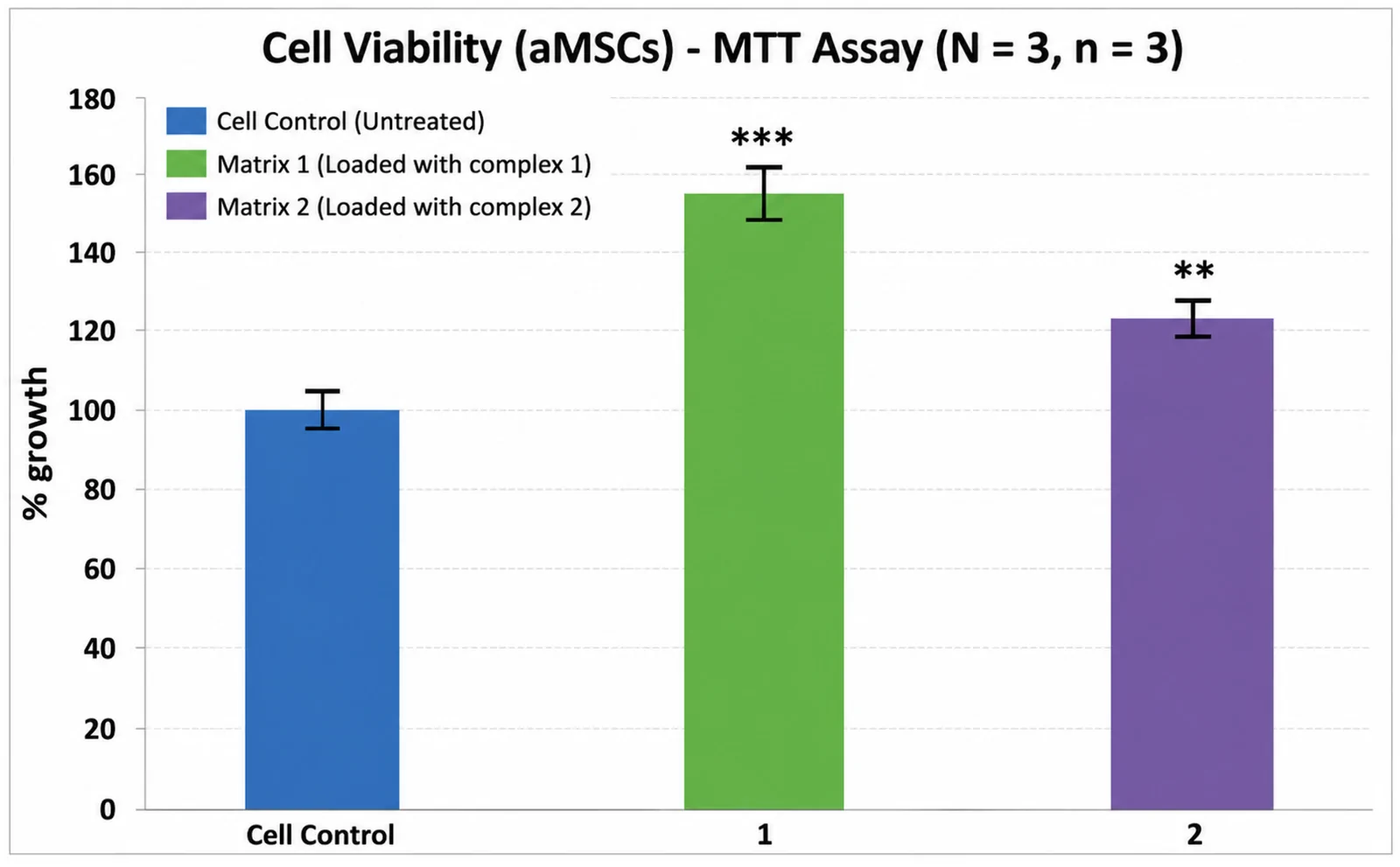
Percentage increase in cell viability of aMSCs after 24 h of exposure to **1** and **2** (1 mg/mL). Values represent the mean ± SD of three independent experiments. Control cell viability was set at 100%. Statistical significance: *p* ≤ 0.01 **, *p* ≤ 0.001 ***.

The MTT assay revealed a significant increase in cell viability in aMSCs treated with both **1** and **2** substrates compared to untreated control cells after 24 h of exposure (Figure 9). Specifically, treatment with substrate **1** resulted in a 156.13% increase in cellular viability relative to control cells (set at 100%), with a high level of statistical significance (*p* ≤ 0.001). Similarly, substrate **2**-treated cells showed a 123.37% increase in viability, also statistically significant (*p* ≤ 0.01). These findings indicate that neither of the silver-loaded hybrid biomaterials exerts cytotoxic effects on mesenchymal stem cells. On the contrary, both substrates actively stimulate and enhance aMSC metabolic activity and proliferation within the 24 h cultivation window.

The observation that silver-functionalized polymer matrices promote stem cell proliferation instead of causing cytotoxicity is very encouraging for regenerative medicine. Multiple interconnected biophysical, biochemical, and structural mechanisms may explain this beneficial cellular response. The underlying carrier matrix is essential for promoting cell survival. A 1:1 blend of PVP and sodium alginate creates a hydrophilic, porous polymeric substrate that supports biological needs. Alginate, a natural polysaccharide, mimics the glycosaminoglycans found in the native extracellular matrix (ECM), providing important biochemical factors for cell anchoring. Meanwhile, the high water-retention capacity of PVP helps to keep the microenvironment well-hydrated. This porous, layered structure, which features interlamellar spaces of up to 300 μm, facilitates cell infiltration and allows for the diffusion of oxygen and nutrients. Additionally, it promotes the formation of focal adhesion complexes, which are important for the initiation of the intracellular signaling pathways that support cell survival and proliferation [41].

High concentrations of free silver ions and silver submicroparticles are toxic due to generating significant oxidative stress. However, when present in very low, controlled concentrations, they can induce a beneficial phenomenon known as hormesis, which occurs with low-dose exposure. At the tested screening concentration of 1mg/mL, our hybrid matrices facilitate a controlled release profile that avoids acute cytotoxicity. The literature suggests that exposure to sub-lethal levels of silver species may trigger transient, low-level reactive oxygen species (ROS) acting as secondary signaling molecules, which in turn have been reported to activate mitogen-activated protein kinase (MAPK) pathways and support cell cycle progression [42,43]. While this represents a plausible literature-backed hypothesis for the pro-proliferative response observed in our assays, we emphasize that specific intracellular signaling cascades were not directly measured in this study. Further concentration-dependent and pathway-blocking experiments will be necessary to confirm whether this mechanism operates in our system. In addition, the exact dose–response relationship should be assessed to confirm whether a classic hormetic curve governs the behavior of the materials developed in the paper.

The higher proliferation rate for substrate **1** (156.13%) compared to substrate **2** (123.37%) is linked to their structural differences and nanoparticle characteristics. As indicated by the SEM analysis, the in situ reduction within the matrix of substrate **1** results in uniform, spherical silver submicroparticles. These spherical submicroparticles have a consistent surface energy profile that is highly compatible with cell membranes. In contrast, the submicroparticles in substrate **2** display a more irregular and non-uniform morphology, which can exert slight physical stress on cell membranes during contact, thereby limiting the extent of the proliferative boost. The release profile of complex **1** involves the dissociation of auxiliary ligands, specifically pyridine and 2-thiophenecarboxylate. These carboxylate-based organic components can act as mild metabolic substrates or cellular signaling molecules. In contrast, complex **2** utilizes bipyridine and nitrate. Although bipyridine is stable, its rigid aromatic structure can occasionally have a very mild cytostatic effect on mammalian cells compared to monocyclic pyridine and biocompatible carboxylates, which slightly reduces the overall proliferation rate of the cells [44,45].

In conclusion, the results from the acute cellular assays show that silver-functionalized PVP/ALG sponges are highly cytocompatible and effectively support the initial attachment and growth of stem cells within 24 h. While the MTT assay provides a precise view of acute metabolic activity and early proliferation, evaluating sustained long-term cytocompatibility remains vital for permanent or bioresorbable implantable matrices. By combining strong, broad-spectrum antimicrobial properties with the ability to enhance mesenchymal stem cell proliferation, these hybrid biomaterials serve as possible candidates for applications in active wound healing, tissue engineering, and bioresorbable medical implants.

### 2.6. Osteogenic Differentiation

**Figure 10:**
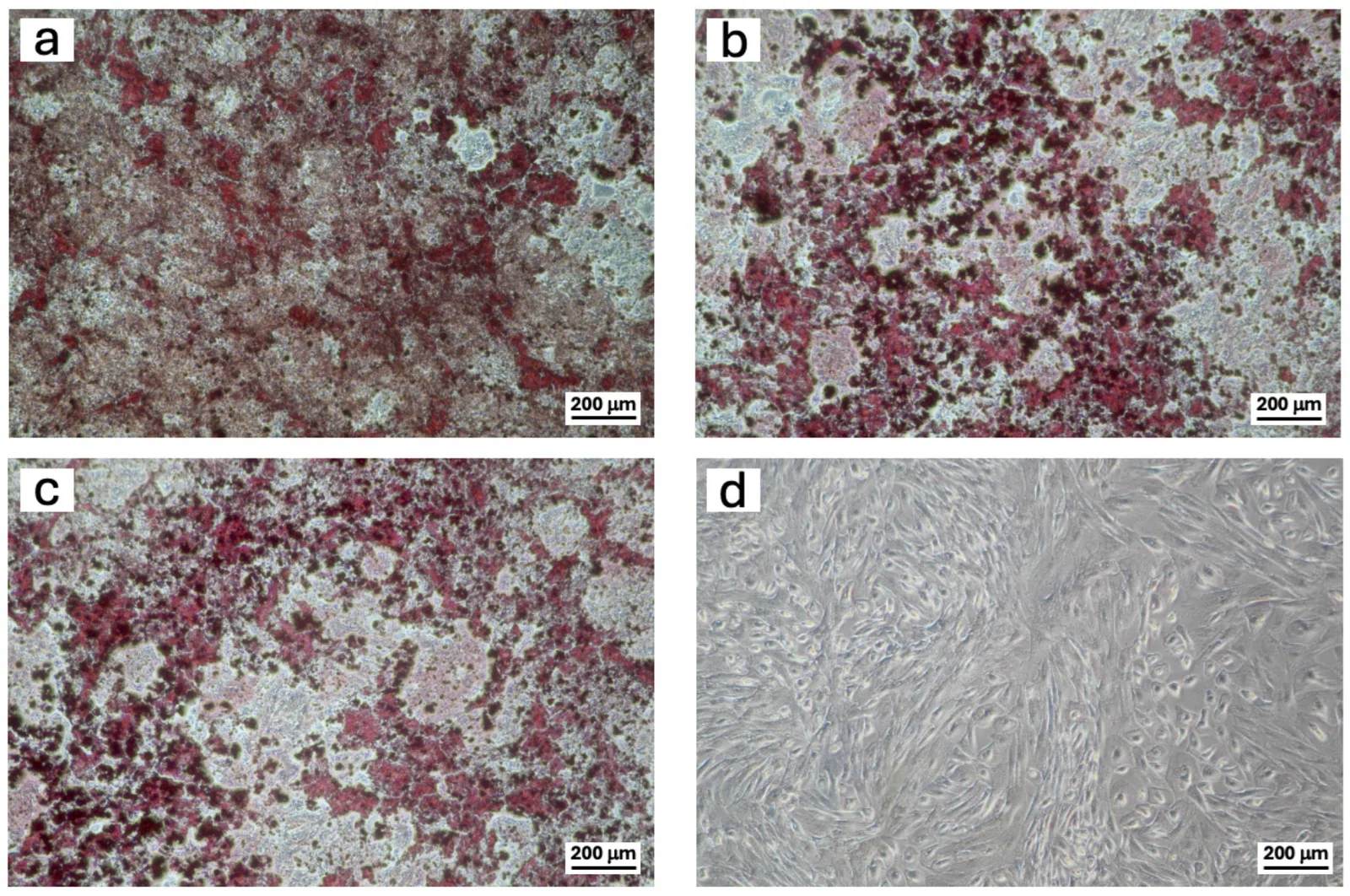
Alizarin Red S staining of aMSCs subjected to osteogenic differentiation. (**a**) **1**-treated cells; (**b**) **2**-treated cells; (**c**) positive control (osteogenic medium alone); and (**d**) negative control (standard growth medium). Images captured using an inverted optical microscope at 10× magnification.

Following a 21-day osteogenic differentiation period, aMSCs treated with the silver-functionalized hybrid substrates **1** and **2** exhibited pronounced matrix mineralization, as visualized by Alizarin Red S staining. Microscopic examination at 10× magnification demonstrated more intense and widespread calcium deposits in the treated groups compared to the positive control (Figure 10). On the other hand, the negative control cells maintained in standard growth medium displayed no signs of mineralization, confirming that the progenitor cells committed to the osteogenic lineage only under osteogenic-inducing conditions.

**Figure 11:**
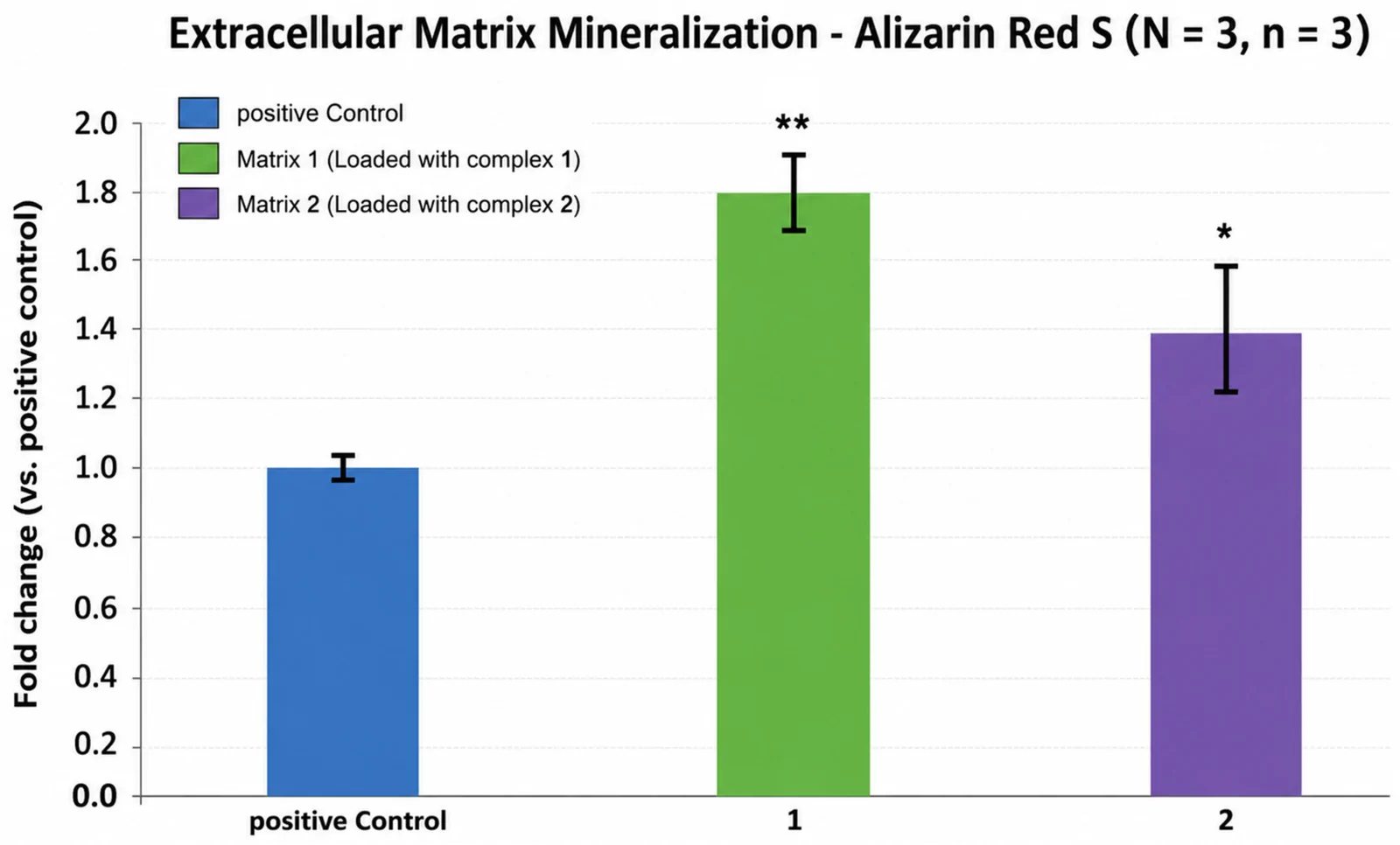
Quantification of Alizarin Red S staining in aMSCs differentiated in the presence of 1 and 2 versus positive control. Data are expressed as relative fold-change ± SD from three independent experiments. Statistical analysis (Dunnett’s test): *p* ≤ 0.05 *; *p* ≤ 0.01 ** versus positive control.

Quantitative analysis of the dissolved calcium deposits further confirmed these visual observations (Figure 11). All osteogenic differentiation and calcium quantification experiments were conducted using N = 3 independent biological replicates, with spectrophotometric readings performed in technical triplicate (n = 3). Data were statistically evaluated using one-way ANOVA followed by Dunnett’s post hoc test relative to the positive control. Cells treated with substrate **1** demonstrated a 1.796-fold increase in mineralization compared to the positive control (*p* ≤ 0.01), while substrate **2** induced a 1.386-fold increase (*p* ≤ 0.05). These results demonstrate a powerful, statistically significant pro-mineralization capacity for both hybrid biomaterials, with substrate **1** exhibiting a noticeably higher efficacy.

The increase in calcium phosphate deposition and matrix mineralization in aMSCs cultured on the silver-loaded polymer matrices can be attributed to the interaction among the physical properties of the PVP/ALG matrix, a prolonged release of silver species, and favorable cellular microenvironments. The biophysical properties of the 1:1 PVP/sodium alginate carrier matrix play an essential dual role in guiding osteogenic matrix deposition. Firstly, the 3D-layered porous structure mimics the trabecular architecture of native bone, creating a supportive mechanical environment that encourages cellular stretching and cytoskeletal tension—mechanical triggers known to promote bone repair over other cell lineages. Secondly, sodium alginate is rich in carboxylate groups. Divalent cations, such as Ba^2+^ used for structural cross-linking, along with the Ca^2+^ ions found in osteogenic differentiation culture media, strongly coordinate with these carboxylate networks. This ion exchange, combined with the localized concentration of divalent ions, creates highly concentrated saturation zones that act as inorganic templates, facilitating the crystallization of hydroxyapatite within the extracellular matrix [46].

The biological impact of silver on bone regeneration is highly dose dependent. While high doses of silver ions (Ag^+^) trigger cell death, the slow release of silver species from the developed hybrid matrices at 1 mg/mL successfully supported enhanced cell viability and matrix mineralization without eliciting cytotoxic degradation. Importantly, the sustained osseopromotive performance and uninterrupted matrix maturation observed over the full 21-day differentiation period serve as functional evidence that continuous exposure to this release profile remains non-cytotoxic to aMSCs. While future work incorporating broad multi-dose gradients and longitudinal live/dead monitoring will be crucial to defining the precise temporal boundaries of this non-toxic therapeutic window, the current data demonstrates a well-tolerated biological profile. Based on the existing literature, low levels of silver ions are reported to potentially stimulate alkaline phosphatase (ALP) activity—the key enzyme responsible for hydrolyzing pyrophosphate and providing free inorganic phosphate for mineralization [47]. At the molecular level, it is hypothesized that controlled silver release may induce a transient, non-lethal emission of intracellular ROS [47], which the literature models describe as secondary triggers capable of activating mitogen-activated protein kinase (MAPK) signaling cascades (e.g., p38 MAPK and ERK1/2) and upregulating downstream osteogenic transcription factors such as RUNX2 and Osterix (OSX) [48]. However, as ALP activity, intracellular ROS levels, and gene/protein expression of RUNX2/OSX were not directly quantified in the present work, these pathways remain hypothetical framework models to guide our ongoing mechanistic investigations.

The enhanced mineralization capacity observed in substrate **1** (with a 1.796-fold increase) compared to substrate **2** (which shows a 1.386-fold increase) aligns well with the findings on substrate microstructural morphology (Section 2.3.2, SEM analysis) and release kinetics (Section 2.3.3). In substrate **1**, the in situ synthesis produces highly uniform, spherical silver submicroparticles that are evenly distributed throughout the porous polymer walls. This high surface area-to-volume ratio of the spherical submicroparticles ensures consistent and optimized contact with the surrounding cellular microenvironment. On the other hand, the irregular and non-uniform submicroparticles in substrate **2** exhibit less predictable surface properties, which may limit their effectiveness in interacting with cell-surface receptors. When solvated, the coordination network of substrate **1** dissociates to release the thiophene-based carboxylate ligand (tph^−^). Carboxylate-containing organic compounds have been shown to act as mild metabolic synergists and osseopromotive support agents, aiding in the complexation and transport of calcium ions across the cell membrane. In contrast, the rigid and highly stable bipyridine ligand in complex **2** remains tightly bound or structurally inert, which does not provide any metabolic or chemical support to the regenerating cells.

Ultimately, these findings show the developed silver-functionalized PVP/ALG matrices are not merely passive biocompatible drug delivery systems. By stimulating high levels of matrix mineralization and cellular proliferation, these hybrid biomaterials function as active osseopromotive and osseoinductive media, making them highly promising candidates for bone tissue engineering, guided bone regeneration, and the coating of orthopedic implants.

## 3. Materials and Methods

### 3.1. General Details

The complexes were synthesized in air using distilled water and commercial solvents: acetonitrile (MeCN, reagent grade, Khimmed, Moscow, Russia), silver(I) acetate (AgOAc, 98%, SRFCHEM, Saint Petersburg, Russia), silver(I) nitrate (AgNO_3_, 99%, Acros), thiophene-2-carboxylic acid (Htph, 98%, Acros Organics, Geel, Belgium), pyridine (py, high-purity grade, Khimmed, Moscow, Russia), 4,4’-bipyridine (bpy, 98%, Alfa Aesar, Haverhill, MA, USA). IR spectra were recorded using a Spectrum 65 FT-IR spectrometer (PerkinElmer Inc., Waltham, MA, USA) equipped with a Quest ATR Accessory (Specac Ltd., Orpington, UK) in the range of 400–4000 cm^−1^. Microprobe analyses were carried out using a Carlo Erba EA 1108 Series CHNS Elemental Analyzer (Carlo Erba Instruments, Milan, Italy) at the Center for Collective Use of the Institute of General and Inorganic Chemistry, Russian Academy of Sciences (IGIC RAS, Moscow, Russia).

### 3.2. Synthesis

#### 3.2.1. Synthesis of [Ag(tph)py_2_]_n_·nHtph (**1**)

A mixture of silver acetate AgOAc (0.084 g, 0.5 mmol) and 2-thiophenecarboxylic acid Htph (0.064 g, 0.5 mmol) was dissolved in 10 mL of an acetonitrile–water mixture (MeCN:H_2_O, 1:1, *v*/*v*). The solution was stirred at 40 °C yielding a transparent mixture. Pyridine (0.080 mL, 1 mmol) was then added, and the resulting clear solution was stirred at 45 °C for an additional 60 min. The solution was filtered and allowed to stand for slow crystallization at room temperature. After 3 days, colorless/white crystals of 1 suitable for single-crystal X-ray diffraction analysis were obtained. The crystals were isolated by decantation, washed with cold MeCN, and dried in air. The yield of **1** was 0.093 g (71%).

FT-IR, ν/cm^−1^: 3070 v.s, 3008 w, 2931 v.w, 2721 v.w, 2457 w, 2321 v.w, 1935 v.w, 1859 v.w, 1690 w, 1597 m, 1516 s, 1484 m, 1442 m, 1393 s, 1259 s, 1217 s, 1148 m, 1099 m, 1058 m, 1033 v.s, 960 s, 930 m, 879 m, 853 m, 755 m, 699 v.s, 668 v.s, 642 v.s, 616 v.s, 504 v.s, 451 v.s, 414 s.

Calc. for C_20_H_17_AgN_2_O_4_S_2_, (%): C 46.08; H 3.29; N 5.37; S 12.30. Found: C 46.26, H 3.30, N 5.51; S 12.43.

#### 3.2.2. Synthesis of [Ag(bpy)]_n_·nNO_3_ (**2**)

Silver nitrate (AgNO_3_, 0.085 g, 0.5 mmol) was dissolved in 10 mL of an acetonitrile-water solvent mixture (MeCN:H_2_O, 1:1, *v*/*v*) at room temperature under stirring. To this transparent solution, 2,2′-bipyridine (Bpy, 0.078 g, 0.5 mmol) dissolved in 5 mL of MeCN was added dropwise. The resulting mixture was stirred at 45 °C for 60 min. Then, the solution was filtered and allowed to slowly crystallize. Crystals of **2** suitable for single-crystal X-ray diffraction analysis were obtained after recrystallization from 7 mL of a 20% aqueous ammonia solution. The resulting crystals were separated from the mother liquor by decantation, washed with cold MeCN, and dried in air. The yield **2** was 0.123 g (75%).

FT-IR, ν/cm^−1^: 3096 br.w, 3046 v.w, 3016 v.w, 2377 v.w, 2050 v.w, 1981 v.w, 1741 v.w, 1660 v.w, 1604 s, 1538 w, 1492 w, 1418 m, 1324 v.w, 1223 s, 1140 w, 1072 w, 1043 w, 1007 w, 976 v.w, 815 v.s, 726 s, 671 v.w, 638 s, 455 s.

Calc. for C_10_H_8_N_3_O_3_Ag, %: C 36.84; H 2.47; N 12.89 Found: C 36.91; H 2.48; N 13.02.

### 3.3. Spectroscopy and Stability ***1*** and ***2***

The UV-Vis spectra were obtained using an SF-2000 UV–Vis spectrophotometer (OKB Spectr LLC, Saint Petersburg, Russia) in aqueous solution across the range of 185–400 nm. The stability of the complexes in solution was monitored by measuring the spectra of the sample (10 μM) over a period of two weeks at room temperature to confirm their chemical integrity during precursor solution processing and scaffold fabrication (the free ligands were measured at ~10^−5^ M to give an absorbance of ~1 for reliable comparison). As noted, this solution-state evaluation reflects material fabrication stability; additional dynamic equilibrium interactions occur upon exposure to complex physiological media containing electrolytes and proteins.

### 3.4. Scanning Electron Microscopy (SEM)

SEM images were taken using a TESCAN AMBER GMH field-emission scanning electron microscope (TESCAN ORSAY HOLDING, Brno, Czech Republic). Images were obtained using an Everhart–Thornley secondary electron (SE) detector at ×3.000–100.000 magnifications and at an accelerating voltage of 20 kV to show silver submicroparticles covered by an organic matrix. SEM images were processed by SEM_scale_bar software (version 5.2, developed by M. Teplonogova; Institute of General and Inorganic Chemistry, Russian Academy of Sciences (IGIC RAS, Moscow, Russia)).

### 3.5. Single-Crystal X-Ray Analysis

X-ray diffraction analysis of complexes **1** and **2** was performed on a Bruker SMART Photon II single-crystal X-ray diffractometer (CCD detector, Mo Kα radiation, λ = 0.71073 Å, graphite monochromator; Bruker AXS Inc., Madison, WI, USA). Semi-empirical adsorption correction was applied using the SADABS program (version 2016/2, Bruker AXS Inc., Madison, WI, USA) [49,50]. The structures were solved by direct methods and refined by full-matrix least squares using the SHELXL program package (George M. Sheldrick, University of Göttingen, Göttingen, Germany) [51] in the anisotropic approximation for non-hydrogen atoms. The hydrogen atom of the OH group in **1** was located in the difference Fourier maps and freely refined without constraints. The positions of the other hydrogen atoms in **1** and **2** were calculated based on stereochemical criteria and refined using the riding model. In structure **1**, the sulfur and carbon atoms of the thiophen cycle are disordered over two positions with nonequal occupancies of 0.831(2)/0.169(2). At the final stage, structure **2** was refined as a two-component twin (twin law is −1 0 0 0 −1 0 1 0 1 and BASF = 0.2136(7)). The main crystallographic data and refinement parameters are summarized in Table 2. The Cambridge Crystallographic Data Centre (CCDC) deposition numbers 2566370 (for **1**) and 2566371 (for **2**) contain the crystallographic data for this paper. These data can be obtained free of charge from CCDC http://www.ccdc.cam.ac.uk.

### 3.6. Assessment of Antimicrobial Activity

The antimicrobial effects of **1** and **2** were tested against five microbial strains: *Escherichia coli* (*E. coli*) ATCC 25922, *Staphylococcus aureus* (*S. aureus*) ATCC 25923, *Pseudomonas aeruginosa* (*P. aeruginosa*) ATCC 27853, *Enterococcus faecalis* (*E. faecalis*) ATCC 29212, and *Candida albicans* (*C. albicans*) ATCC 10231 from the American Type Culture Collection (ATCC, Manassas, VA, USA). All tested bacterial and fungal reference strains are classified as Risk Group 2 agents requiring Biosafety Level 2 (BSL-2) containment, and all antimicrobial procedures were performed in a certified BSL-2 facility adhering to standard biosafety protocols [50]. Microorganisms were cultured in Brain Heart Infusion (BHI) broth (Difco Laboratories, BD Diagnostic Systems, Sparks, MD, USA) at 37 °C for bacteria and 28 °C for *C. albicans*, in the presence of sterile substrates at 1 mg/mL for 24 h. Microbial growth was assessed by measuring optical density (OD) at 600 nm with the BioPhotometer spectrophotometer (Eppendorf AG, Hamburg, Germany), and results were compared to untreated control cultures grown in BHI alone.

### 3.7. Isolation and Culture of Adipose-Derived Mesenchymal Stromal Cells

Adipose-derived mesenchymal stromal cells (aMSCs) were isolated from subcutaneous adipose tissue obtained from a 12-month-old female sheep immediately post-mortem (collected as a byproduct from an authorized commercial slaughterhouse following standard agricultural processing). Primary ovine cell cultures were handled under Biosafety Level 1 (BSL-1) standards [50]. The adipose tissue was rinsed thoroughly with sterile physiological saline solution to remove blood and potential contaminants. Subsequently, the tissue was minced into small fragments using sterile surgical scissors and enzymatically digested with Collagenase type IA (Sigma-Aldrich Co., Ltd., Gillingham, Dorset, UK) for 60 min at 37 °C under constant agitation. Following enzymatic digestion, the cell suspension was filtered and centrifuged at 800× *g* for 10 min. The resulting cell pellet was resuspended in Dulbecco’s Modified Eagle Medium (DMEM; Life Technologies Ltd., Paisley, UK), supplemented with 10% fetal bovine serum (FBS; Life Technologies Ltd., Paisley, UK), and 1% Penicillin–Streptomycin (100 U/mL Penicillin and 100 µg/mL Streptomycin; Sigma-Aldrich Co., Ltd., Gillingham, Dorset, UK), and 2.5 µg/mL Amphotericin B (Sigma-Aldrich Co., Ltd., Gillingham, Dorset, UK). Cells were seeded into tissue culture flasks and incubated at 37 °C in a humidified atmosphere containing 5% CO_2_ until reaching approximately 80–85% confluence.

### 3.8. MTT Assay for Cell Viability

At passage 3, aMSCs were trypsinized and seeded into 6-well plates at a density of 20,000 cells/mL. After 24 h to allow cell adhesion, the culture medium was replaced with fresh medium containing the biomaterials **1** or **2**, each at a concentration of 1 mg/mL. The substrates were previously sterilized by autoclaving at 121 °C for 20 min in dry form. Following autoclaving, the crosslinked PVP/ALG substrates maintained their macroscopic 3D structural integrity and porous matrix shape without thermal deformation or physical breakdown. Control wells received only the standard growth medium without any substrates. After a further 24 h of incubation, cell viability was assessed using the 3-(4,5-Dimethylthiazol-2-yl)-2,5-diphenyltetrazolium bromide (MTT; Sigma-Aldrich Co., Ltd., Gillingham, Dorset, UK) assay. Cells were incubated with MTT solution (0.5 mg/mL in DMEM) for 3 h at 37 °C. The resulting formazan crystals, formed by mitochondrial dehydrogenase activity in viable cells, were solubilized in Isopropanol (Sigma-Aldrich Co., Ltd., Gillingham, Dorset, UK). Absorbance was measured at 600 nm (a standard detection wavelength within the broad 550–600 nm absorption band of solubilized formazan) using a BioPhotometer spectrophotometer (Eppendorf AG, Hamburg, Germany), and results were expressed as a percentage relative to control cells. In addition, an Eppendorf BioPhotometer uses pre-calibrated filter/wavelength channels, with 600 nm as the standard for photometric and turbidity applications. Because both control and treated samples were read under identical optical path and wavelength settings (600 nm), any minor shift in absolute extinction coefficient cancels out completely during normalization. Therefore, the relative statistical differences, assay sensitivity, and biological conclusions remain fully accurate, robust, and directly comparable.

### 3.9. Osteogenic Differentiation of aMSCs

For osteogenic induction, aMSCs were seeded into 6-well plates at a density of 40,000 cells/mL. After 24 h, the culture medium was replaced with osteogenic induction medium composed of DMEM supplemented with 50 µg/mL ascorbic acid, 10 mM β-glycerophosphate, and 10^−7^ M dexamethasone (all Sigma-Aldrich, Merck KGaA, Darmstadt, Germany). Experimental groups were treated with either 1 or 2 (1 mg/mL), which remained present throughout the 21-day differentiation period. The positive control group received only the osseoinductive medium, while the negative control group was maintained in standard growth medium (DMEM supplemented with 10% FBS, 1% Penicillin–Streptomycin, and 2.5 µg/mL Amphotericin B, without osteogenic supplements). At the end of the differentiation period, calcium deposition was assessed via Alizarin Red S staining (Sigma-Aldrich Co., Ltd., Gillingham, Dorset, UK). Cells were fixed in 4% paraformaldehyde (Sigma-Aldrich Co., Ltd., Gillingham, Dorset, UK) for 30 min at room temperature, then incubated with 3% Alizarin Red S in isopropanol for 30 min. To quantify mineralization, stained cells were treated with 5% sodium lauryl sulfate (Sigma-Aldrich Co., Ltd., Gillingham, Dorset, UK) in 0.5 N hydrochloric acid (HCl) (Sigma-Aldrich Co., Ltd., Gillingham, Dorset, UK) for 30 min, and absorbance was read at 490 nm using a BioPhotometer spectrophotometer (Eppendorf AG, Hamburg, Germany).

### 3.10. Statistical Analysis

All experiments were conducted in triplicate and repeated independently. Data were analyzed using Dunnett’s post hoc test to compare treated groups with their respective controls. Statistical significance was considered at *p* ≤ 0.05 *, *p* ≤ 0.01 **, and *p* ≤ 0.001 ***. Statistical analyses were performed using JMP Pro software (version 14, SAS Institute Inc., Cary, NC, USA).

## 4. Conclusions

In this study, highly advanced, multifunctional biomaterials based on porous crosslinked polyvinylpyrrolidone/sodium alginate sponges functionalized separately with active silver(I) complexes (**1** and **2**) have been developed and comprehensively characterized. These hybrid constructs successfully combine localized bacteriostatic surface protection with promising cell-supportive and matrix-mineralizing capabilities, making them promising materials for further exploration in the treatment of bone tissue damage and guided bone regeneration.

The crosslinked PVP/ALG substrates functionalized separately with complex **1** or **2** (each loaded at a concentration of 1 mg/mL) demonstrated excellent in vitro biocompatibility, as evidenced by enhanced cellular viability and facilitated late-stage extracellular matrix mineralization in aMSCs. The observed increase in cell viability (up to 156.13% for substrate **1** and a highly supportive profile for substrate **2**) suggests a potential pro-proliferative effect driven by the biocompatible crosslinked polymer network and controlled silver release. Furthermore, the pronounced Alizarin Red S staining and quantitative analysis of calcium deposition confirmed a strong osseopromotive efficacy for both materials, with the spherical, structurally uniform silver submicroparticles in substrate **1** eliciting a superior 1.796 -fold increase in matrix mineralization compared to the **1**.386-fold increase induced by substrate **2**. This enhanced mineralization is attributed to a combination of scaffold microstructural features, calcium-coordinating alginate groups, and well-tolerated silver release profiles that support sustained cellular activity.

Moreover, both crosslinked substrates exhibited moderate yet statistically significant bacteriostatic and anti-biofilm activity broadly across tested microbial species—including Gram-positive and Gram-negative species as well as fungi—indicating that early protection is largely independent of Gram classification. This controlled 20–35% microbial growth inhibition achieved at 1 mg/mL highlights a well-balanced therapeutic window designed to mitigate initial bacterial adherence and biofilm formation without exerting cytotoxic side effects on regenerating progenitor cells. When evaluating these outcomes, the complex nature of nutrient-rich biological media (containing competing proteins and chloride anions that partially bind free Ag^+^ ions) represents an important factor influencing net silver bioavailability. This dual functionality—supporting tissue regeneration outcomes while simultaneously providing localized bacteriostatic surface protection—positions complexes **1** and **2** as promising candidates for biomedical applications, particularly as active coatings for orthopedic implants and bioresorbable tissue scaffolds. These findings, validated by robust statistical analysis (N = 3), provide a strong foundation for future in-depth mechanistic, transcriptomic, and in vivo translational evaluations under extended, dynamic physiological media environments aimed at optimizing the therapeutic use of **1** and **2** complexes in bone defect repair.

## Figures and Tables

**Table 1:** Parameters of the main bond lengths (Å) in **1** and **2**.

Bond Length	1	2
Ag(1)…O(1)	2.8701(15)	2.739(2)
Ag(1)…O(2)	2.7900(13) ^1,2^	2.732(3) ^3^
Ag(1)–N	2.2027(15)	2.140(2)–2.144(2)

^1^ _-1+X,+Y,+Z_; ^2^ _1-X,-Y,1-Z_; ^3^ _1/2-X,1/2+Y,1/2-Z_.

**Table 2:** Selected crystal data and parameters for structure refinement of the title compounds **1** and **2**.

Complex	1	2
Empirical formula	C_20_H_17_AgN_2_O_4_S_2_	C_10_H_8_N_3_O_3_Ag
Formula weight	521.34	326.06
*T* (K)	150	100
Crystal system Space group	Monoclinic *P*2_1_/*c*	Monoclinic *C*2/*c*
*a* (Å)	6.2217(4)	12.7304(7)
*b* (Å)	8.7372(5)	9.8321(6)
*c* (Å)	18.8566(12)	18.3336(12)
*α* (deg)	90	90
*β* (deg)	94.482(2)	110.0290(17)
*γ* (deg)	90	90
*V* (Å^3^)	1021.91(11)	2156.0(2)
*Z*	2	8
*D*_calc_ (g·cm^−3^)	1.694	2.009
*μ* (mm^−1^)	1.220	1.869
*F* (000)	524.0	1280.0
Reflections measured	17698	16548
Independent reflections	2976	3126
*R*_int_	0.0409	0.0402
*R*_1_ (all data)	0.0370	0.0249
*wR*_2_ (all data)	0.0598	0.0566
*R*_1_ (*I* > 2*σ*(*I*))	0.0283	0.0243
*wR*_2_ (*I* > 2*σ*(*I*))	0.0555	0.0562
*GooF*	1.107	1.105
Δ*ρ*_max_/*ρ*_min_ (e/Å^3^)	0.36/−0.47	0.62/−0.85

## Data Availability

The raw data supporting the findings of this study are available from the corresponding authors upon reasonable request.
